# Epidemiology and health outcomes associated with hyperkalemia in a primary care setting in England

**DOI:** 10.1186/s12882-019-1250-0

**Published:** 2019-03-06

**Authors:** Laura Horne, Akhtar Ashfaq, Sharon MacLachlan, Marvin Sinsakul, Lei Qin, Robert LoCasale, James B. Wetmore

**Affiliations:** 1 Global Medical Affairs, AstraZeneca, Gaithersburg, MD USA; 2 Real World Evidence, Evidera, London, UK; 3 Health Economics and Payer Analytics, AstraZeneca, Gaithersburg, MD USA; 4 Real World Evidence, AstraZeneca, Gaithersburg, MD USA; 5 Chronic Disease Research Group and Division of Nephrology, Hennepin County Medical Center, Minneapolis, MN USA

**Keywords:** Chronic kidney disease, Healthcare resource utilization, Hyperkalemia, Incidence

## Abstract

**Background:**

Real-world incidence, clinical consequences, and healthcare resource utilization (HRU) of hyperkalemia (HK) remain poorly characterized, particularly in patients with specific comorbidities.

**Methods:**

Data from the Clinical Practice Research Datalink and Hospital Episode Statistics databases were analyzed to determine incidence of an index HK event, subsequent clinical outcomes, and HRU in the English population. Factors associated with index HK in a primary care setting were also identified for those with an index HK event during the study period (2009–2013) and matched controls.

**Results:**

The overall incidence rate of an index HK event was 2.9 per 100 person-years. Use of renin–angiotensin–aldosterone system inhibitors was strongly associated with HK (odds ratio, 13.6–15.9). Few patients (5.8%) had serum potassium (K^+^) retested ≤ 14 days following the index event; among those retested, 32% had HK. Following an index HK event, all-cause hospitalization, HK recurrence, and kidney function decline were the most common outcomes (incidence rates per 100 person-years: 14.1, 8.1, and 6.7, respectively), with higher rates in those with comorbidities or K^+^ > 6.0 mmol/L. Mortality and arrhythmia rates were higher among those with K^+^ > 6.0 mmol/L. Older age, comorbid diabetes mellitus, and mineralocorticoid receptor antagonist use were associated with HK recurrence. Relatively few patients received testing or prescriptions to treat HK following an event.

**Conclusions:**

Severe index HK events were associated with adverse outcomes, including arrhythmia and mortality. Despite this, retesting following an index event was uncommon, and incidence of recurrence was much higher than that of the index event.

**Electronic supplementary material:**

The online version of this article (10.1186/s12882-019-1250-0) contains supplementary material, which is available to authorized users.

## Background

Hyperkalemia (HK) is a potentially life-threatening electrolyte abnormality typically characterized by elevated serum potassium (K^+^) levels (> 5.0 mmol/L) [1]. It is often associated with severe adverse clinical outcomes, including cardiac arrhythmias and mortality, with increasing frequency as K^+^ increases [1, 2]. Generally, dietary restrictions are recommended to control K^+^, and while there are a few acute and chronic drug therapies available to help control K^+^, these are not widely prescribed and there may be a need for improved treatments. [3]. Sodium polystyrene sulfonate, the traditional first-line oral therapy, has not been robustly tested in clinical trials for HK, has a variable onset of effect, and is associated with gut necrosis when administered over prolonged periods [4, 5]. Patiromer, a K^+^-binding agent recently approved in the United States and Europe, is a potential option for long-term use but must not be taken within 3 h of other oral medications [6], potentially limiting its use. With few options for long-term control of K^+^ and little consensus on how and when to treat, patients with HK may be undertreated, thereby increasing their risk of HK-related adverse clinical outcomes.

Few studies have evaluated HK incidence or prevalence; those published primarily describe highly selected patient populations. Frequency estimates of HK vary based on study designs, including definition of HK and frequency of K^+^ monitoring, and patient characteristics (including comorbidities and medication use) [7, 8]. For example, the HK prevalence among patients with heart failure (HF) in clinical trials evaluating renin–angiotensin–aldosterone system antagonists ranged from 1.4 to 6.0% depending on the definition of HK used, severity of HF, and medications used [9]. In two studies of patients with chronic kidney disease (CKD), the prevalence of HK, defined as K^+^ ≥ 5.5 mmol/L, ranged from 3.2 to 31.5% [7, 8].

Availability of high-quality electronic health records enables quantification of HK incidence and the clinical conditions, use of medications, and real-world health consequences associated with HK in the general population. Here, we report results from a population-based analysis characterizing the incidence rate of HK and its associated factors, frequency of retesting, clinical consequences, and healthcare resource utilization (HRU) associated with HK among patients receiving healthcare in England.

## Methods

### Study design and data sources

Retrospective analyses, as described in detail below, were conducted using data from the outpatient/primary care Clinical Practice Research Datalink (CPRD) database linked to the inpatient Hospital Episode Statistics (HES) and the Office for National Statistics (ONS) databases. The CPRD is an electronic database of anonymous longitudinal medical records for > 11 million individuals from ~ 700 primary care practices across the United Kingdom [10]. This database captures information such as patient demographics, prescription drug usage, clinical events, laboratory tests, specialist referrals, and hospital admissions and their major outcomes [10]. Independent Scientific Advisory Committee approval (protocol number: 16_217R2) was obtained from the CPRD. The HES database provides information on inpatient care provided at National Health Service hospitals in England [11]. The ONS database contains mortality-related information, including cause of death, for all deaths registered in England and Wales [12].

A cohort analysis was used to determine HK incidence rate, incidence rates and factors associated with negative clinical outcomes following an index HK event, frequency of HK retesting after the index HK event, and HRU associated with HK. Clinical outcomes of interest included HK recurrence, cardiac arrhythmia, cardiac arrest, HF, acute kidney injury, decline in kidney function, dialysis treatment, all-cause hospitalization, and all-cause mortality. Retesting was evaluated over the 14-day period immediately following the date of the index HK episode. HRU in the 30 days following an index HK event was characterized by the number of laboratory tests, hospitalizations, outpatient visits, specialist referrals, and prescribed medications. Demographics and clinical factors associated with the index HK event were identified using a case-control analysis.

### Patient population

Adults (aged ≥ 18 years) with an index HK event between January 1, 2009, and December 31, 2013, were identified from the linked CPRD/HES database. The index HK event was defined by at least one of the following: a READ diagnosis code, a laboratory result of K^+^ ≥ 5.0 mmol/L in the CPRD, or an International Classification of Diseases, Tenth Revision (ICD-10) diagnosis code in the HES. Patients were excluded from the study for any of the following: an index K^+^ ≥ 10.0 mmol/L, < 365 days of observation time between the date of the HK event and the current registration or up to standard dates, history of HK events before study start (January 1, 2009), active cancer, or recent history of volume depletion or dehydration (based on a READ diagnosis code). Only patients with ≥ 1 visit to a general practitioner within the year were included.

### Study definitions

Severity of index HK was classified as follows: K^+^ 5.0 to ≤ 5.5 mmol/L or CPRD diagnosis code with no corresponding laboratory result, K^+^ > 5.5 to ≤ 6.0 mmol/L, or K^+^ > 6.0 mmol/L or ICD-10 code for HK in the HES database, regardless of K^+^ level (for simplicity, subsequent text will refer to numeric levels). READ and ICD-10 codes used in this analysis are shown in Additional file 1 Table S1. Baseline estimated glomerular filtration rate (eGFR) was calculated using the Modification of Diet in Renal Disease formula [13], using the most recent value for serum creatinine in the 365 days preceding the index HK event. HK recurrence was defined as a second event of elevated K^+^ at any time, provided there was a return to normal K^+^ (< 5.0 mmol/L). HF, cardiac arrest, and cardiac arrhythmias were defined using ICD-10 codes in the secondary care setting (e.g. HES). Declining kidney function was defined as the presence of diagnostic codes and/or eGFR showing a decline from baseline CKD stage in the CPRD or HES. Acute kidney injury was defined using READ or ICD-10 codes. Provision of dialysis was identified by the presence of a diagnostic or procedure code in the CPRD or HES. All-cause mortality was determined using the date of death in the linked ONS database, with the date preceding the end of study as defined in the CPRD. All-cause hospitalization was determined based on patient record in the HES. For each patient for each event, follow-up began on the day after the index HK event and continued until the earliest of the following: clinical event of interest, patient transferred out of the practice, death, or end of study period (December 31, 2013).

### Statistical analyses

The incidence rate of an index HK event and each designated clinical outcome was determined for the overall population, stratified by prespecified clinical subgroups (patients with diabetes mellitus, CKD, HF, or hypertension) and index HK event severity. Crude incidence rates were calculated as the number of patients with the designated outcome divided by the total number seeking care and were not adjusted for baseline differences across subgroups. The frequency of HK retesting within 14 days of the index HK event was assessed.

This study used both case-control and cohort analyses to address different study objectives. First, a case-control analysis was conducted to identify factors associated with an index HK event. Each patient with an index HK event in the primary or secondary care setting was eligible to serve as a case. Up to four controls were selected from the CPRD/HES database for each HK case and matched on the care setting of visit (i.e. CPRD or HES), presence of at least one laboratory test of any type on the visit date (CPRD matches only), visit date (± 3 months), age (± 3 years), time since registration (± 4 years), and sex. Conditional logistic regression analyses were used to evaluate relationships between potential risk factors and HK; adjusted odds ratios (ORs) and 95% confidence intervals (CIs) are reported. Additionally, a cohort analysis was conducted to identify factors associated with designated clinical outcomes. In the cohort analyses, Cox proportional hazards models were generated to evaluate risk of clinical outcomes and variables identified as significant in the stepwise analysis are presented as adjusted hazard ratios with 95% CIs, adjusting for all other variables in the model.

Descriptive analysis was performed to evaluate HRU outcomes at 3, 7, and 30 days following an index HK event for the overall population and by HK severity stratum. One inpatient admission, one outpatient visit, all laboratory measurements, and all prescriptions filled for any drug could be included per day.

## Results

A total of 195,178 patients with an index HK event during the study period were analyzed (Additional file 1: Figure S1). Patient demographics and baseline characteristics are shown in Table 1. Most patients were female (52.1%), with a mean (standard deviation [SD]) age of 60.6 (16.6) years and mean (SD) eGFR of 80.5 (21.1) mL/min/1.73 m^2^. Common baseline comorbidities included hypertension, hyperlipidemia, ischemic heart disease, CKD, and obstructive lung disease. Patients with an index HK event with K^+^ > 6.0 mmol/L had generally higher frequencies of comorbidities, especially ischemic heart disease, arrhythmia, and atrial fibrillation, compared with patients with an index HK event with K^+^ ≤ 6.0 mmol/L, although eGFR did not appear to be substantially lower.

**Table 1:** Patient demographics and baseline characteristics

	Overall (*N* = 195,178)	Serum K^+^ level during index HK event, mmol/L		
		5.0 to ≤ 5.5 (*n* = 177,945)^a^	> 5.5 to ≤ 6.0 (*n* = 14,020)	> 6.0 (*n* = 3213)^b^
Age, years	60.6 ± 16.6	60.5 ± 16.5	60.7 ± 17.0	63.7 ± 18.7
Female	101,700 (52.1)	92,847 (52.2)	7174 (51.2)	1679 (52.3)
BMI, kg/m^2^	28.3 ± 6.1	28.4 ± 6.1	27.9 ± 6.1	27.8 ± 6.7
eGFR, mL/min/1.73 m^2^	80.5 ± 21.1	80.6 ± 20.9	79.7 ± 22.0	78.2 ± 23.9
Smoking status				
Never	100,511 (51.5)	91,977 (51.7)	6933 (49.5)	1606 (50.0)
Current	36,852 (18.9)	33,212 (18.7)	2962 (21.1)	679 (21.1)
Former	57,042 (29.2)	52,089 (29.3)	4059 (28.9)	888 (27.6)
Unknown	773 (0.4)	667 (0.4)	66 (0.5)	40 (1.2)
Comorbidities				
Hypertension	98,860 (50.7)	90,156 (50.7)	6839 (48.8)	1865 (58.1)
Hyperlipidemia	38,245 (19.6)	35,009 (19.7)	2525 (18.0)	711 (22.1)
Ischemic heart disease	24,886 (12.8)	22,486 (12.6)	1735 (12.4)	665 (20.7)
Myocardial infarction	10,064 (5.2)	9061 (5.1)	703 (5.0)	300 (9.3)
Arrhythmia (including atrial fibrillation)	18,475 (9.5)	16,513 (9.3)	1364 (9.7)	598 (18.6)
Atrial fibrillation	13,182 (6.8)	11,653 (6.6)	1033 (7.4)	496 (15.4)
Heart failure	4354 (2.2)	3716 (2.1)	349 (2.5)	289 (9.0)
Cerebrovascular disease	12,620 (6.5)	11,241 (6.3)	964 (6.9)	415 (12.9)
Peripheral artery disease	3531 (1.8)	3154 (1.8)	265 (1.9)	112 (3.5)
Diabetes (types 1 and 2)	24,323 (12.5)	22,203 (12.5)	1649 (11.8)	471 (14.7)
Chronic kidney disease	34,912 (17.9)	31,560 (17.7)	2457 (17.5)	895 (27.9)
Obstructive lung disease	35,734 (18.3)	32,405 (18.2)	2607 (18.6)	722 (22.5)
Liver disease	7483 (3.8)	6600 (3.7)	586 (4.2)	297 (9.2)
RAAS inhibitor use				
Never	126,475 (64.8)	115,215 (64.8)	9405 (67.1)	1855 (57.7)
Current	59,465 (30.5)	54,532 (30.7)	4004 (28.6)	929 (28.9)
Former	9238 (4.7)	8198 (4.6)	611 (4.4)	429 (13.4)
Concomitant medication				
ACE inhibitor	44,000 (22.5)	40,367 (22.7)	2989 (21.3)	644 (20.0)
ARB	15,495 (7.9)	14,253 (8.0)	991 (7.1)	251 (7.8)
MRA	3909 (2.0)	3336 (1.9)	395 (2.8)	178 (5.5)
Loop diuretic	11,493 (5.9)	10,126 (5.7)	962 (6.9)	405 (12.6)
Thiazide diuretic	14,204 (7.3)	13,008 (7.3)	927 (6.6)	269 (8.4)
NSAID	18,049 (9.3)	16,492 (9.3)	1309 (9.3)	248 (7.7)
Antibiotic	3068 (1.6)	2652 (1.5)	309 (2.2)	107 (3.3)

Data are given as mean ± standard deviation or n (%)

^a^Or Clinical Practice Research Datalink diagnosis code in the absence of laboratory results

^b^Or Hospital Episode Statistics diagnosis code, regardless of serum K^+^ level

*ACE* angiotensin-converting enzyme, *ARB* angiotensin II receptor blocker, *BMI* body mass index, *eGFR* estimated glomerular filtration rate, *HK* hyperkalemia, *K*^*+*^ potassium, *MRA* mineralocorticoid receptor antagonist, *NSAID* nonsteroidal anti-inflammatory drug, *RAAS* renin–angiotensin–aldosterone system

Concomitant medications were similar across HK severity strata; key exceptions were loop diuretics and mineralocorticoid receptor antagonists (MRAs), which were roughly two-fold more common among patients with an index HK event with K^+^ > 6.0 mmol/L versus K^+^ ≤ 6.0 mmol/L.

### Incidence rates and factors associated with hyperkalemia

**Fig. 1:**
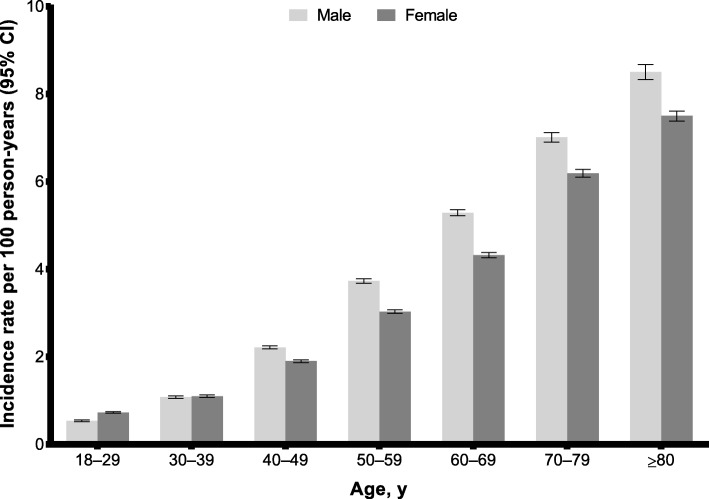
Incidence of index hyperkalemia event based on age and sex. Error bars show the 95% confidence interval (CI)

The overall incidence rate of an index HK event was 2.86 per 100 person-years (95% CI, 2.83–2.89) (Table 2). Most patients experienced an index HK event with K^+^ 5.0 to ≤ 5.5 mmol/L (91.2%), of which 61.0% had an event with K^+^ between 5.0 and 5.1 mmol/L. The proportion of patients who had an HK event with K^+^ 5.5 to ≤ 6.0 mmol/L and K^+^ > 6.0 mmol/L was 7.2 and 1.6%, respectively. The HK incidence rate tended to increase with age, regardless of sex (Fig. 1). Similar age-related trends were observed when patients were stratified by HK severity (Additional file 1: Figure S2).

**Table 2:** Incidence of index hyperkalemic event

	Number of patients with index HK	Incidence of index HK event per 100 person-years (95% CI)
Overall	195,178	2.86 (2.83–2.89)
Serum K^+^ level during HK event, mmol/L		
5.0 to ≤ 5.5 or CPRD diagnosis code in the absence of laboratory results	177,945	2.61 (2.58–2.63)
> 5.5 to ≤ 6.0	14,020	0.21 (0.20–0.21)
> 6.0 or HES diagnosis code, regardless of serum K^+^ level	3213	0.05 (0.04–0.05)

*CI* confidence interval, *CPRD* Clinical Practice Research Datalink, *HES* Hospital Episode Statistics, *HK* hyperkalemia, *K*^*+*^ potassium

Factors associated with the index HK event are shown in Table 3. Younger age was associated with increased odds of HK, while use of angiotensin-converting enzyme (ACE) inhibitors, angiotensin II receptor blockers (ARBs), and MRAs, as well as presence of a baseline eGFR value, was strongly associated with the development of HK.

**Table 3:** Factors associated with the index hyperkalemic event

	Odds ratio (95% CI)	
	Unadjusted	Stepwise adjusted
Age range, years		
18–29	REF	REF
30–39	1.07 (1.04–1.10)	0.86 (0.83–0.89)
40–49	1.15 (1.12–1.19)	0.68 (0.66–0.71)
50–59	1.41 (1.37–1.45)	0.68 (0.66–0.70)
60–69	1.60 (1.56–1.65)	0.67 (0.65–0.69)
70–79	1.76 (1.72–1.81)	0.60 (0.58–0.62)
≥ 80	1.77 (1.73–1.83)	0.62 (0.59–0.64)
Sex		
Male	REF	REF
Female	0.89 (0.88–0.90)	1.08 (1.07–1.10)
Presence of measured baseline laboratory values		
BUN	1.12 (1.05–1.19)	0.70 (0.66–0.75)
eGFR	31.49 (30.51–32.52)	29.81 (28.84–30.80)
Smoking status		
Current	1.05 (1.04–1.15)	1.17 (1.15–1.18)
Former	1.40 (1.39–1.26)	1.26 (1.24–1.27)
Never	REF	REF
Unknown	0.72 (0.66–1.28)	1.32 (1.20–1.44)
Comorbidity		
Hypertension	2.29 (2.27–2.32)	0.88 (0.87–0.90)
Hyperlipidemia	1.42 (1.41–1.44)	0.85 (0.84–0.87)
Ischemic heart disease	1.63 (1.61–1.66)	0.85 (0.83–0.87)
Arrhythmia (including atrial fibrillation)	1.33 (1.31–1.36)	0.91 (0.89–0.93)
Heart failure	1.94 (1.87–2.02)	0.89 (0.84–0.94)
Cerebrovascular disease	1.31 (1.29–1.34)	0.93 (0.91–0.96)
Peripheral artery disease	1.71 (1.64–1.78)	1.13 (1.08–1.19)
Diabetes (types 1 and 2)	1.97 (1.94–2.01)	0.95 (0.93–0.97)
Chronic kidney disease	1.69 (1.67–1.71)	1.04 (1.02–1.06)
Obstructive lung disease	0.92 (0.91–0.93)	0.95 (0.93–0.96)
Liver disease	1.13 (1.10–1.17)	0.93 (0.90–0.96)
Concomitant medication		
ACE inhibitor	15.11 (14.80–15.43)	13.63 (13.31–13.95)
ARB	14.56 (14.05–15.09)	15.89 (15.27–16.54)
MRA	17.47 (16.17–18.88)	7.77 (7.06–8.54)
Antibiotics	0.32 (0.31–0.33)	0.33 (0.32–0.34)
Loop diuretics	2.79 (2.72–2.86)	1.39 (1.34–1.44)
Thiazide diuretics		
Bendroflumethiazide	1.86 (1.82–1.90)	0.85 (0.83–0.88)
Indapamide	1.45 (1.36–1.54)	0.57 (0.52–0.62)
Hydrochlorothiazide	2.46 (2.26–2.67)	0.83 (0.75–0.93)

*ACE* angiotensin-converting enzyme, *ARB* angiotensin II receptor blocker, *BUN* blood urea nitrogen, *CI* confidence interval, *eGFR* estimated glomerular filtration rate, *MRA* mineralocorticoid receptor antagonist, *REF* reference value

### Hyperkalemia retesting

Overall, only 5.8% of patients with an index HK event had K^+^ retested within 14 days of the index event (Table 4). Patients with an index HK event with K^+^ > 6.0 mmol/L were retested more frequently than those whose index event was K^+^ 5.0 to ≤ 5.5 mmol/L or K^+^ > 5.5 to ≤ 6.0 mmol/L (55.3, 3.9, and 23.4%, respectively). Among patients retested within 14 days, 32.0% had a second HK event with an elevated K^+^, but this varied only slightly by index K^+^ level: 36.8% of patients whose index HK event was K^+^ > 6.0 mmol/L had elevated K^+^ upon retesting compared with 29.5% of patients with an index HK event with K^+^ 5.0 to ≤ 5.5 mmol/L. Furthermore, a repeat HK event with K^+^ > 6.0 mmol/L was identified in 1.7% of those with an index HK event with K^+^ 5.0 to ≤ 5.5 mmol/L and in 19.2% of those with an index HK event with K^+^ > 6.0 mmol/L.

**Table 4:** Hyperkalemia retesting

	Overall (*N* = 194,035)	Serum K^+^ level during index HK event, mmol/L		
		5.0 to ≤ 5.5 (*n* = 177,945)^a^	> 5.5 to ≤ 6.0 (*n* = 14,020)	> 6.0 (*n* = 2070)^b^
Patients who had retest within 14 days, n (%)	11,342 (5.8)	6922 (3.9)	3276 (23.4)	1144 (55.3)
Patients with HK after retest	3632 (32.0)	2045 (29.5)	1166 (35.6)	421 (36.8)
5.0 to ≤ 5.5 mmol/L^a^	–	1782 (87.1)	876 (75.1)	242 (57.5)
> 5.5 to ≤ 6.0 mmol/L	–	228 (11.1)	249 (21.4)	98 (23.3)
> 6.0 mmol/L^b^	–	35 (1.7)	41 (3.5)	81 (19.2)

^a^Or Clinical Practice Research Datalink diagnosis code in the absence of laboratory results

^b^Or Hospital Episode Statistics diagnosis code, regardless of serum K^+^ level

*HK* hyperkalemia, *K*^*+*^ potassium

### Clinical outcomes following an index hyperkalemic event

The incidence rate of clinical outcomes after an index HK event was higher among patients with a more severe index HK event compared with those with a less severe index event. The largest absolute difference in incidence rates among those with an index HK event with K^+^ > 6.0 mmol/L versus K^+^ 5.0 to ≤ 5.5 mmol/L, respectively, was observed for all-cause mortality (12.57 [95% CI, 11.63–13.56] vs 2.51 [95% CI, 2.46–2.56]) and all-cause hospitalization (28.93 [95% CI, 27.22–30.72] vs 13.86 [95% CI, 13.73–13.99]) (Table 5). Incidence rates of renal-associated outcomes at these respective K^+^ levels also differed, with higher rates observed among those with more severe index HK: decline in kidney function (14.61 [95% CI, 13.46–15.83] vs 6.54 [95% CI, 6.45–6.62]), acute kidney injury (4.09 [95% CI, 3.52–4.72] vs 1.18 [95% CI, 1.15–1.22]), and dialysis (1.55 [95% CI, 1.23–1.94] vs 0.12 [95% CI, 0.11–0.13]). Rates of the most studied clinical outcomes in patients with diabetes or CKD were approximately double those of the overall study population and were ~ 3- to 6-fold higher in patients with HF compared with the overall study population.

**Table 5:** Incidence rates per 100 person-years (95% confidence interval) of clinical outcomes following index hyperkalemic event

	Overall	Serum K^+^ level during index HK event, mmol/L			Comorbidity			
		5.0 to ≤ 5.5^a^	> 5.5 to ≤ 6.0	> 6.0^b^	Diabetes	CKD	HF	Hypertension
HK recurrence	8.07 (7.98–8.16)	7.82 (7.73–7.91)	10.68 (10.28–11.08)	12.14 (11.14–13.21)	17.17 (16.76–17.59)	15.33 (15.02–15.65)	20.98 (19.83–22.18)	11.12 (10.97–11.27)
HF	0.61 (0.59–0.64)	0.60 (0.58–0.63)	0.64 (0.55–0.74)	1.38 (1.07–1.75)	1.18 (1.08–1.28)	1.57 (1.48–1.66)	–	0.94 (0.90–0.98)
Cardiac arrhythmia	1.07 (1.03–1.10)	1.05 (1.02–1.09)	1.13 (1.01–1.26)	1.83 (1.46–2.27)	1.58 (1.46–1.70)	2.47 (2.35–2.60)	5.06 (4.33–5.88)	1.57 (1.52–1.63)
Cardiac arrest	0.14 (0.13–0.15)	0.13 (0.12–0.15)	0.17 (0.12–0.22)	0.44 (0.28–0.66)	0.28 (0.23–0.33)	0.31 (0.27–0.35)	0.80 (0.61–1.03)	0.20 (0.18–0.22)
Decline in kidney function	6.68 (6.60–6.76)	6.54 (6.45–6.62)	7.36 (7.04–7.69)	14.61 (13.46–15.83)	12.09 (11.75–12.43)	9.69 (9.45–9.93)	16.57 (15.56–17.64)	8.75 (8.62–8.89)
Acute kidney injury	1.26 (1.23–1.30)	1.18 (1.15–1.22)	1.87 (1.71–2.03)	4.09 (3.52–4.72)	2.71 (2.56–2.87)	4.00 (3.85–4.16)	7.15 (6.49–7.85)	1.94 (1.88–2.00)
Dialysis	0.14 (0.13–0.15)	0.12 (0.11–0.13)	0.21 (0.16–0.27)	1.55 (1.23–1.94)	0.31 (0.27–0.37)	0.41 (0.36–0.46)	0.57 (0.41–0.77)	0.20 (0.19–0.22)
All-cause mortality	2.73 (2.68–2.78)	2.51 (2.46–2.56)	3.83 (3.61–4.05)	12.57 (11.63–13.56)	4.31 (4.13–4.50)	7.09 (6.90–7.29)	14.76 (13.91–15.65)	3.85 (3.76–3.93)
All-cause hospitalization	14.14 (14.01–14.27)	13.86 (13.73–13.99)	15.53 (15.03–16.03)	28.93 (27.22–30.72)	17.6 (17.18–18.03)	22.37 (21.98–22.77)	43.17 (41.33–45.07)	16.24 (16.05–16.43)

^a^Or Clinical Practice Research Datalink diagnosis code in the absence of laboratory results

^b^Or Hospital Episode Statistics diagnosis code, regardless of serum K^+^ level

*CKD* chronic kidney disease, *HF* heart failure, *HK* hyperkalemia, *K*^*+*^ potassium

Demographics and clinical factors associated with HK recurrence and other adverse clinical outcomes are shown in Table 6. After age, the presence of comorbid diabetes and use of MRAs at the time of the index HK event were associated with the highest ORs for recurrent HK (1.86 [95% CI, 1.81–1.91] and 1.74 [95% CI, 1.64–1.85], respectively); ACE inhibitor and ARB use at the time of the index HK event was also associated with recurrent HK. Having an index HK event with K^+^ > 6.0 mmol/L was associated with all studied outcomes, most strongly with mortality, given that patients with an index HK event with K^+^ > 6.0 mmol/L had 3.3-fold higher odds of death than patients with an index HK event with K^+^ 5.0 to ≤ 5.5 mmol/L.

**Table 6:** Factors associated with hyperkalemia recurrence and other adverse clinical outcomes following an index hyperkalemic event

	Stepwise-adjusted odds ratio (95% CI)			
	HK recurrence	Cardiac arrhythmia	All-cause hospitalization	Death
Age range, years				
18–29	REF	REF	REF	REF
30–39	1.30 (1.13–1.48)	1.08 (0.58–2.02)	0.96 (0.90–1.02)	1.58 (0.92–2.71)
40–49	1.85 (1.64–2.08)	1.52 (0.88–2.62)	0.90 (0.85–0.95)	3.14 (1.95–5.08)
50–59	2.29 (2.04–2.58)	2.94 (1.74–4.96)	0.98 (0.93–1.04)	6.19 (3.87–9.90)
60–69	2.73 (2.43–3.07)	6.83 (4.08–11.43)	1.15 (1.09–1.22)	13.36 (8.39–21.27)
70–79	3.23 (2.87–3.64)	15.52 (9.28–25.96)	1.42 (1.34–1.50)	30.23 (19.00–48.11)
≥ 80	3.45 (3.06–3.89)	33.18 (19.82–55.56)	1.77 (1.67–1.88)	91.03 (57.21–144.86)
Sex				
Male	REF	REF	REF	REF
Female	–	0.70 (0.65–0.75)	–	0.87 (0.83–0.90)
Baseline laboratory values				
BUN	–	–	1.50 (1.37–1.64)	–
eGFR	0.99 (0.99–0.99)	1.00 (1.00–1.00)	1.00 (1.00–1.00)	1.00 (1.00–1.00)
Smoking status				
Current	1.12 (1.08–1.15)	1.22 (1.10–1.34)	1.18 (1.15–1.21)	1.86 (1.76–1.96)
Former	1.07 (1.04–1.10)	1.04 (0.97–1.11)	1.09 (1.06–1.11)	1.07 (1.02–1.11)
Never	REF	REF	REF	REF
Unknown	1.04 (0.84–1.28)	1.29 (0.79–2.12)	0.99 (0.85–1.16)	1.91 (1.53–2.37)
Comorbidity				
Hypertension	1.23 (1.19–1.27)	1.11 (1.03–1.19)	–	–
Hyperlipidemia	1.04 (1.01–1.07)	0.86 (0.80–0.93)	–	0.79 (0.76–0.83)
Ischemic heart disease	1.15 (1.11–1.18)	1.42 (1.31–1.54)	1.28 (1.25–1.31)	1.12 (1.07–1.17)
Arrhythmia (including atrial fibrillation)	1.10 (1.06–1.14)	–	1.30 (1.27–1.34)	1.33 (1.27–1.39)
Heart failure	1.10 (1.03–1.17)	1.32 (1.12–1.56)	1.14 (1.08–1.20)	1.38 (1.29–1.48)
Cerebrovascular disease	1.04 (1.00–1.08)	1.27 (1.16–1.40)	1.18 (1.14–1.22)	1.60 (1.53–1.68)
Peripheral artery disease	1.10 (1.03–1.17)	1.29 (1.11–1.50)	1.31 (1.25–1.38)	1.43 (1.32–1.54)
Diabetes (types 1 and 2)	1.86 (1.81–1.91)	–	1.08 (1.05–1.11)	1.27 (1.21–1.33)
Chronic kidney disease	1.19 (1.15–1.23)	–	1.08 (1.05–1.11)	1.09 (1.05–1.15)
Obstructive lung disease	1.06 (1.03–1.09)	1.36 (1.26–1.46)	1.28 (1.25–1.31)	1.36 (1.31–1.42)
Liver disease	1.21 (1.15–1.28)	1.47 (1.26–1.72)	1.50 (1.44–1.56)	2.01 (1.87–2.17)
Concomitant medication				
ACE inhibitor	1.27 (1.23–1.31)	–	0.90 (0.88–0.92)	0.77 (0.74–0.80)
ARB	1.16 (1.12–1.21)	–	0.92 (0.89–0.95)	0.66 (0.62–0.70)
MRA	1.74 (1.64–1.85)	1.36 (1.15–1.61)	1.15 (1.10–1.22)	1.23 (1.14–1.32)
Loop diuretics	1.10 (1.06–1.15)	1.78 (1.61–1.97)	1.33 (1.28–1.38)	1.87 (1.78–1.97)
Bendroflumethiazide	0.90 (0.86–0.93)	1.17 (1.06–1.30)	0.96 (0.92–0.99)	–
Indapamide	0.84 (0.75–0.94)	–	–	–
NSAID	1.21 (1.17–1.25)	–	1.30 (1.27–1.34)	–
Antibiotics	–	–	1.57 (1.48–1.66)	1.50 (1.38–1.65)
Serum K^+^ level during index HK event, mmol/L				
5.0 to ≤ 5.5^a^	–	–	–	–
> 5.5 to ≤ 6.0	1.39 (1.33–1.44)	1.07 (0.96–1.21)	1.10 (1.06–1.13)	1.41 (1.33–1.50)
> 6.0^b^	1.34 (1.23–1.47)	1.47 (1.18–1.83)	1.63 (1.54–1.74)	3.27 (3.02–3.54)

^a^Or Clinical Practice Research Datalink diagnosis code in the absence of laboratory results

^b^Or Hospital Episode Statistics diagnosis code, regardless of serum K^+^ level

*ACE* angiotensin-converting enzyme, *ARB* angiotensin II receptor blocker, *BUN* blood urea nitrogen, *CI* confidence interval, *eGFR* estimated glomerular filtration rate, *HK* hyperkalemia, *K*^*+*^ potassium, *MRA* mineralocorticoid receptor antagonist, *NSAID* nonsteroidal anti-inflammatory drug, *REF* reference value

### Healthcare resource utilization

**Fig. 2:**
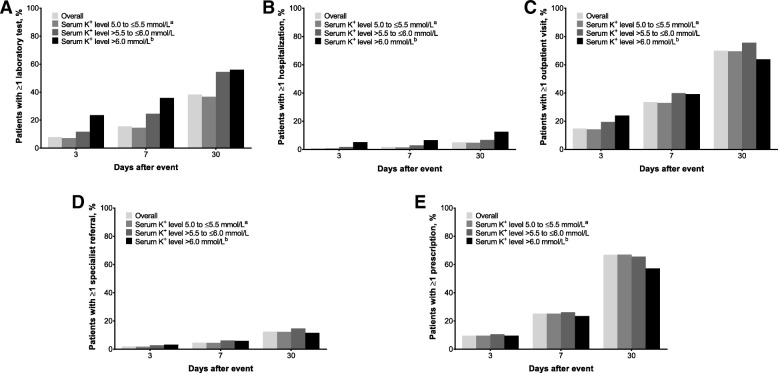
Healthcare resource utilization following an index hyperkalemia event in overall population and by hyperkalemia severity. (**a**) Proportion of patients with ≥ 1 laboratory test; (**b**) proportion of patients with ≥ 1 hospitalization; (**c**) proportion of patients with ≥ 1 outpatient visit; (**d**) proportion of patients with ≥ 1 specialist referral; and (**e**) proportion of patients with ≥ 1 prescription. ^a^ Or Clinical Practice Research Datalink diagnosis code in the absence of laboratory results. ^b^ Or Hospital Episode Statistics diagnosis code, regardless of serum potassium (K^+^) level

In general, the proportion of patients with ≥ 1 reported HRU following the index HK event increased with severity of the index HK event, with differences apparent as early as 3 days afterward (Fig. 2). Among patients with ≥ 1 reported HRU, the mean number of laboratory tests increased with the severity of the index HK event, yet only 14.5% of patients with an index HK event with K^+^ 5.0 to ≤ 5.5 mmol/L had any follow-up laboratory tests within a week, compared with 24.5% of patients with an index HK event with K^+^ > 5.5 to ≤ 6.0 mmol/L. Furthermore, only 35.8% of patients with an index HK event with K^+^ > 6.0 mmol/L had any laboratory tests within a week. Similarly, the mean number of hospitalizations generally increased with HK severity at each time point examined. However, HK severity did not appear to be associated with outpatient visits, specialist referrals, or prescriptions.

## Discussion

Using a large comprehensive medical records database from England, we found most index HK events involved only modest increases in K^+^ levels; however, severe HK occurred more often than might be expected within the general population. Furthermore, HK appeared to be persistent upon retesting and had a graded association with adverse outcomes including hospitalization and death, yet rarely resulted in rapid retesting, even among those with a severe index HK event.

To our knowledge, this is the first population-based study to evaluate the incidence of an index HK event, patterns of retesting K^+^, and subsequent clinical outcomes in a large general primary-care setting. The observed incidence rate of an index HK event in this study (~ 3 per 100 person-years) was generally comparable to that of other studies. A Swedish observational study using a marginally more strict definition of HK (K^+^ > 5.0 mmol/L) found a crude incidence rate of HK of 5 per 100 person-years [14]. This finding could be due to differences in our study populations, frequency of testing, use of concomitant medications, or prevalence of comorbidities.

Several key factors were associated with index HK in our study. The presence of a baseline eGFR value was strongly associated with HK, likely reflecting prescribers’ awareness of its inherent risk associated with kidney dysfunction.

Additionally, use of ACE inhibitors, ARBs, and MRAs, each known to potentially increase K^+^ [14–16], were found to be strong risk factors for HK. Of note, more than half of individuals who had HK were not users of RAAS inhibitors, which may indicate that providers judged these patients to be at disproportionate risk of developing HK, and so did not prescribe these drugs.

In contrast, some characteristics appeared inversely related to index HK development. Interestingly, increased age appeared to reduce the likelihood of an index HK event in this study; the exact reason for this is unknown. Thiazide diuretics, as anticipated, were associated with a lower risk of index HK [17, 18]; loop diuretics, in contrast, were associated with an increased risk of HK. While the nature of our study cannot determine why this is the case, the explanation for this finding may be that the patients with the most complex medical conditions or who were judged by their medical providers as being most at risk of HK were also the ones most likely to receive loop, as opposed to thiazide, diuretics.

Strikingly, retesting of K^+^ within 2 weeks of an index HK event was uncommon. While this may be because most index HK events involved only mildly elevated K^+^, retesting was far less common than might be expected. Retesting was uncommon even in patients with an index HK event with K^+^ > 6.0 mmol/L, with only 55% retested within 2 weeks of an index HK event. In addition, among those with an index HK event with K^+^ > 6.0 mmol/L who were retested, roughly one in five had K^+^ > 6.0 mmol/L when retested within 2 weeks following the event. This is suggestive of both an underappreciation of the importance of K^+^ retesting, especially when K^+^ levels approach or even exceed 6.0 mmol/L, and the likelihood of sustained HK [14]. The lack of prompt K^+^ retesting and the persistence of frank HK could possibly place patients at undue risk of major clinical events. The incidence rate of HK recurrence was particularly high among patients with diabetes, CKD, HF, or hypertension, suggesting that clinicians should pay particular attention to these at-risk groups.

Hospitalization was correlated with the severity of HK. While not all HRU markers were elevated following an HK episode, HK appeared to be associated with hospitalization over short (3 days) and intermediate (30 days) lengths of time. Although the direction of causality is uncertain, HK can be a sign of acute illness (rather than a cause of it), which may portend HK as a particular risk for impending illness and hospitalization, and underscore the need to closely monitor patients with an index HK event. Combined with a comparative lack of HK retesting and high rate of HK recurrence after retesting (especially when the index HK event had K^+^ > 6.0 mmol/L), these findings suggest that prudent HK testing, follow-up, and retesting might identify a patient subgroup at undue risk for adverse events who could benefit from timely interventions in response to HK.

Several study design and database limitations should be considered when interpreting these findings. The CPRD captures data among patients seeking healthcare in a primary care setting and may not fully represent the healthcare status of all patients; this study may not be generalizable to populations outside of England because of inherent differences in populations, treatment strategies, and services. Although the CPRD/HES database is well validated [10, 19], the potential for misclassified or nonspecific ICD-10 codes, diagnosis codes, and/or laboratory data exists and may lead to over- or under-reporting of HK events and other clinical outcomes, especially because clinical diagnoses were not verified by chart review. One study found the sensitivity of ICD-10 codes for diagnosing HK to be low (14.6% at hospital admission), which may contribute to under-reporting of HK events [20]. However, the likely effect of this would be to underestimate the risk associated with HK. As noted earlier, laboratory data are not available in the HES database; therefore, if severe cases were not coded correctly, they would have been excluded from this analysis. Incidence of HK may also be misreported because this analysis primarily relied on laboratory findings of HK in the CPRD which likely included instances of spurious HK due to hemolysis. Further, the data sources could not account for previously unobserved occurrences of HK which might have obfuscated comorbidities at baseline, as it may be unclear if these conditions are a result of untreated HK. In addition, this analysis did not include emergency care data, which may contribute to potential undercapturing of HK diagnoses given that repeat testing may have occurred in an emergency setting. The incidence of repeat testing may have been further affected by mis-diagnoses and inaccurate K^+^ levels due to hemolysis. The analysis may also be limited because of infrequent measurements of K^+^. Finally, this study cannot assess whether there is a causal relationship between HK and outcomes following HK events.

## Conclusions

This study is the first to quantify HK incidence, K^+^ testing patterns, and outcomes associated with HK in a primary care setting in England, and findings suggest that current attention to HK in general practice may be suboptimal. Although patients who experienced an index HK event, particularly with K^+^ > 6.0 mmol/L, were more likely to experience a second occurrence of HK as well as adverse clinical outcomes, repeat testing was not common, and it is unclear whether directed treatment for HK was administered. While causality could not be determined in this study, an association between HK and adverse clinical outcomes was identified, suggesting that underdiagnosis and undertreatment of HK may increase the risk of adverse clinical outcomes. As well-tolerated therapies for the long-term management of HK become readily available, the benefits of more frequent testing of K^+^ and restoring normokalemia may become apparent. More research is needed in at-risk patients with frequently monitored K^+^ levels whose K^+^ is controlled to normal levels to fully understand HK and its consequences.

## Additional file

Additional file 1: **Table S1.** Codes used to define adverse events in this analysis. **Figure S1.** Selection of patients for inclusion in the study. **Figure S2.** Hyperkalemia incidence according to age and sex based on (A) serum potassium (K^+^) 5.0 to ≤ 5.5 mmol/L or Clinical Practice Research Datalink diagnosis code in the absence of laboratory results, (B) serum K^+^ > 5.5 to ≤ 6.0 mmol/L, and (C) serum K^+^ > 6.0 mmol/L or Hospital Episode Statistics diagnosis code, regardless of serum K^+^ level in the initial hyperkalemic event. (PDF 159 kb)

## Abbreviations

ACE: Angiotensin-converting enzyme
ARBs: Angiotensin II receptor blockers
CI: Confidence interval
CKD: Chronic kidney disease
CPRD: Clinical Practice Research Datalink
eGFR: Estimated glomerular filtration rate
HES: Hospital Episode Statistics
HF: Heart failure
HK: Hyperkalemia
HRU: Healthcare resource utilization
ICD-10: International Classification of Diseases, Tenth Revision
K^+^: Potassium
MRAs: Mineralocorticoid receptor antagonists
ONS: Office for National Statistics
OR: Odds ratio
SD: Standard deviation
