# Supplementary material for: Epidemiology and health outcomes associated with hyperkalemia in a primary care setting in England
Source: BMC Nephrol. 2019 Mar 6;20:85. doi: 10.1186/s12882-019-1250-0 (PMC6404265; doi:10.1186/s12882-019-1250-0)
Supplement: Supplementary file 1 — Table S1. Codes used to define adverse events in this analysis. Figure S1. Selection of patients for inclusion in the study. Figure S2. Hyperkalemia incidence according to age and sex based on (A) serum potassium (K+) 5.0 to ≤ 5.5 mmol/L or Clinical Practice Research Datalink diagnosis code in the absence of laboratory results, (B) serum K+ > 5.5 to ≤ 6.0 mmol/L, and (C) serum K+ > 6.0 mmol/L or Hospital Episode Statistics diagnosis code, regardless of serum K+ level in the initial hyperkalemic event. (PDF 159 kb) [file 12882_2019_1250_MOESM1_ESM.pdf]

Additional File 1

Table S1 Codes used to define adverse events in this analysis

Outcome	Description	Code		
		**ICD-10**	**OPCS-4**	**READ**
Dialysis	Care involving dialysis	Z49		
	Dependence on renal dialysis	Z99.2		
	Extracorporeal dialysis	Z49.1		
	Injury due to failure of sterile precautions during kidney dialysis or other perfusion	Y62.2		
	Injury due to foreign object accidentally left in body during kidney dialysis or other perfusion	Y61.2		
	Injury due to unintentional cut, puncture, perforation, or hemorrhage during kidney dialysis or other perfusion	Y60.2		
	Insertion or removal of peritoneal dialysis catheter		x41.x	
	Kidney dialysis as the cause of abnormal reaction of the patient, or of later complication, without mention of misadventure at the time of the procedure	Y84.1		
	Mechanical complication of vascular dialysis catheter	T82.4		
	Other dialysis	Z49.2		
	Placement of apparatus for compensation for renal failure		x42.x	
	Preparatory care for dialysis	Z49.0		
	Renal dialysis		x40.x	
	Peritoneal dialysis sample			4I29.00
	Compensation for renal failure			7L1A.00
	Dialysis for renal failure			7L1A.11
	Renal dialysis			7L1A000
	Thomas intravascular shunt for dialysis			7L1A011
	Peritoneal dialysis			7L1A100
	Hemodialysis NEC			7L1A200
	Hemofiltration			7L1A300
	Automated peritoneal dialysis			7L1A400
	Continuous ambulatory peritoneal dialysis			7L1A500
	Peritoneal dialysis NEC			7L1A600
	Hemoperfusion			7L1A700
	Other specified compensation for renal failure			7L1Ay00
	Compensation for renal failure NOS			7L1Az00
	Extracorporeal albumin hemodialysis			7L1f000
	[X] Peritoneal dialysis associated peritonitis			SP05613
	Kidney dialysis with complication, without blame			TB11.00
	Renal dialysis with complication, without blame			TB11.11
	[V] Renal dialysis status			ZV45100
	[V] Aftercare involving intermittent dialysis			ZV56.00
	[V] Aftercare involving extracorporeal dialysis			ZV56000
	[V] Aftercare involving renal dialysis NOS			ZV56011

Outcome	Description	Code		
		ICD-10	OPCS-4	READ
	[V] Preparatory care for dialysis			ZV56100
	[V] Other specified aftercare involving intermittent dialysis			ZV56y00
	[V] Aftercare involving peritoneal dialysis			ZV56y11
	[V] Unspecified aftercare involving intermittent dialysis			ZV56z00
	[X] Other dialysis			ZVu3G00

ICD-10 International Classification of Diseases, Tenth Revision, *NEC* not elsewhere classified, *NOS* not otherwise specified, *OPCS-4* OPCS Classification of Interventions and Procedures version 4

Figure S1 Selection of patients for inclusion in the study.

CPRD Clinical Practice Research Datalink, *HES* Hospital Episode Statistics, *HK* hyperkalemia, K^+ potassium, *UTS* up to standard

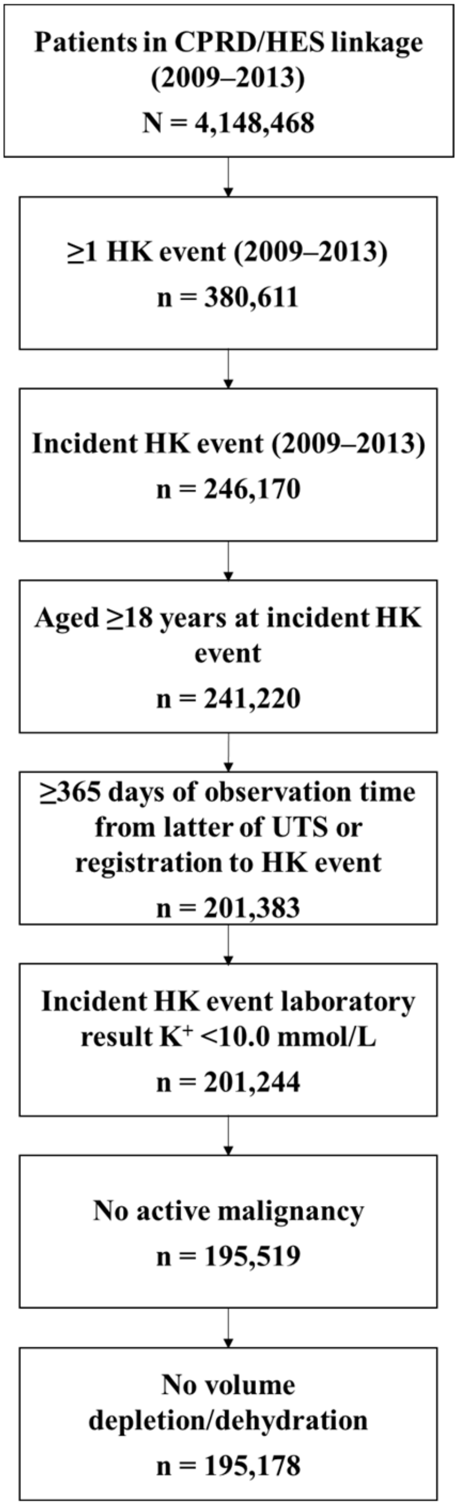

Figure S2 Hyperkalemia incidence according to age and sex based on (A) serum potassium (K^+) 5.0 to ≤ 5.5 mmol/L or Clinical Practice Research Datalink diagnosis code in the absence of laboratory results, (B) serum K^+ > 5.5 to ≤ 6.0 mmol/L, and (C) serum K^+ > 6.0 mmol/L or Hospital Episode Statistics diagnosis code, regardless of serum K^+ level in the initial hyperkalemic event. Error bars are the 95% confidence interval (CI)

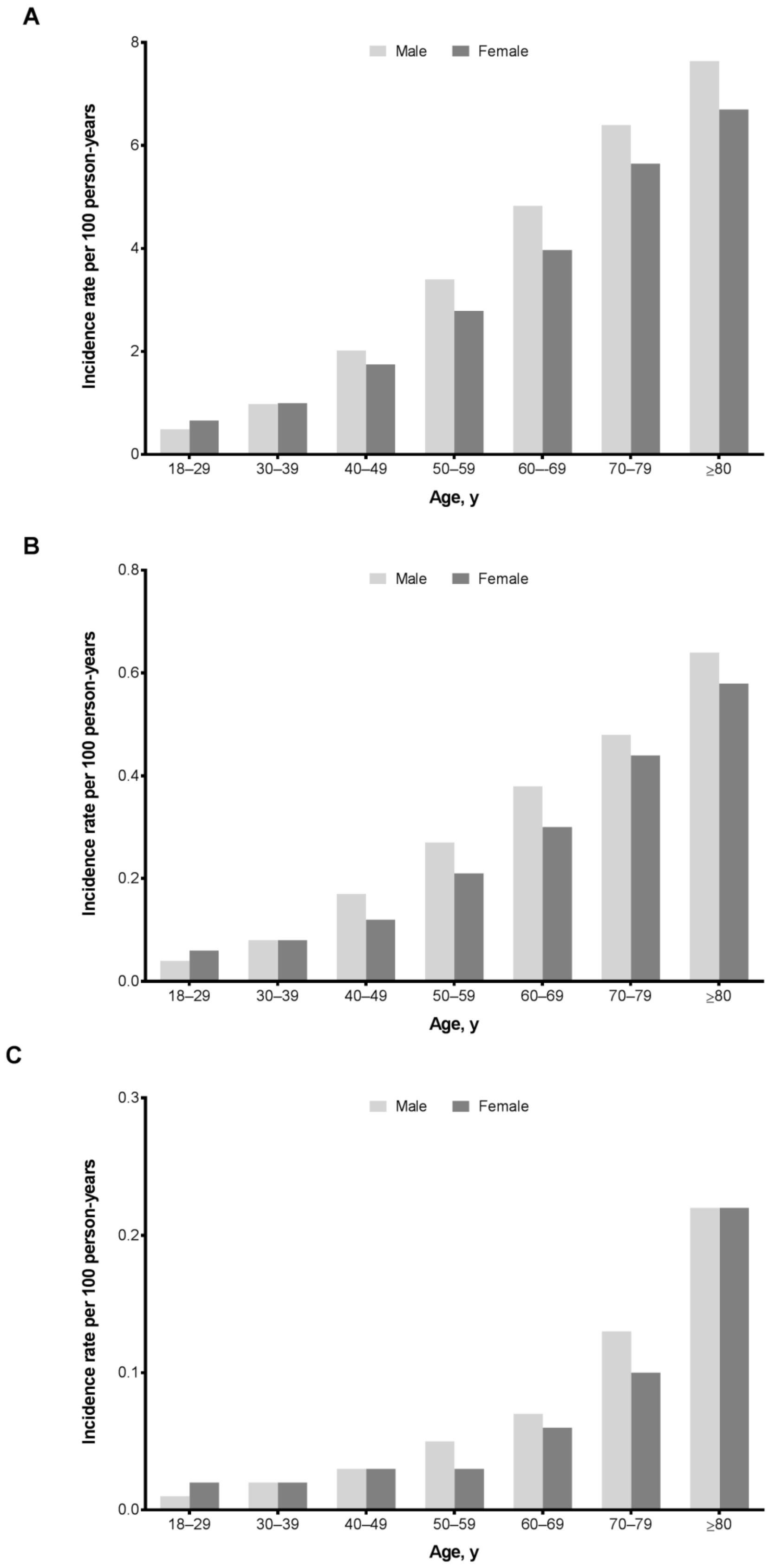
A
B
C
Male
Female
Incidence rate per 100 person-years
Age, y
18–29
30–39
40–49
50–59
60–69
70–79
≥80
0
2
4
6
8
0.0
0.2
0.4
0.6
0.8
0.1
0.3
